# Rnf220 is Implicated in the Dorsoventral Patterning of the Hindbrain Neural Tube in Mice

**DOI:** 10.3389/fcell.2022.831365

**Published:** 2022-03-24

**Authors:** Yu-Bing Wang, Ning-Ning Song, Lei Zhang, Pengcheng Ma, Jia-Yin Chen, Ying Huang, Ling Hu, Bingyu Mao, Yu-Qiang Ding

**Affiliations:** ^1^ Key Laboratory of Arrhythmias, Ministry of Education, East Hospital, Department of Anatomy and Neurobiology, Tongji University School of Medicine, Shanghai, China; ^2^ Department of Laboratory Animal Science, Fudan University, Shanghai, China; ^3^ State Key Laboratory of Medical Neurobiology, MOE Frontiers Center for Brain Science, Institutes of Brain Science, Fudan University, Shanghai, China; ^4^ State Key Laboratory of Genetic Resources and Evolution, Kunming Institute of Zoology, Chinese Academy of Sciences, Kunming, China

**Keywords:** dorsoventral patterning, hindbrain, Rnf220, serotonergic neuron, oligodendrocyte

## Abstract

Rnf220 is reported to regulate the patterning of the ventral spinal neural tube in mice. The brainstem has divergent connections with peripheral and central targets and contains unique internal neuronal groups, but the role of Rnf220 in the early development of the hindbrain has not been explored. In this study, *Nestin*-Cre-mediated conditional knockout (*Rnf220* ^Nestin^ CKO) mice were used to examine if *Rnf220* is involved in the early morphogenesis of the hindbrain. *Rnf220* showed restricted expression in the ventral half of ventricular zone (VZ) of the hindbrain at embryonic day (E) 10.5, and as development progressed, *Rnf220*-expressing cells were also present in the mantle zone outside the VZ at E12.5. In *Rnf220* ^Nestin^ CKO embryos, alterations of progenitor domains in the ventral VZ were observed at E10.5. There were significant reductions of the p1 and p2 domains shown by expression of *Dbx1*, Olig2, and Nkx6.1, accompanied by a ventral expansion of the Dbx1^+^ p0 domain and a dorsal expansion of the Nkx2.2^+^ p3 domain. Different from the case in the spinal cord, the Olig2^+^ pMN (progenitors of somatic motor neuron) domain shifted and expanded dorsally. Notably, the total range of the ventral VZ and the extent of the dorsal tube were unchanged. In addition, the post-mitotic cells derived from their corresponding progenitor domain, including oligodendrocyte precursor cells (OPCs) and serotonergic neurons (5-HTNs), were also changed in the same trend as the progenitor domains do in the CKO embryos at E12.5. In summary, our data suggest similar functions of Rnf220 in the hindbrain dorsoventral (DV) patterning as in the spinal cord with different effects on the pMN domain. Our work also reveals novel roles of Rnf220 in the development of 5-HTNs and OPCs.

## Introduction

Ubiquitination is an important post-translational modification in eukaryotic cells, involved in a variety of cellular processes, including signal transduction and transcriptional regulation (Pickart, 2001; Pickart and Eddins, 2004; Mukhopadhyay and Riezman, 2007). The entire course of ubiquitination is catalyzed by the ubiquitin-activating enzyme, the ubiquitin-conjugating enzyme and the ubiquitin ligase (E3), successively (Morreale and Walden, 2016). There are about 600 E3 ubiquitin ligases in human, and Rnf220 is a newly discovered one of them (Deshaies and Joazeiro, 2009).

As a member of the RING finger protein family, Rnf220 is highly conserved among different species. Rnf220 was first identified as an E3 ubiquitin ligase in 2010, which interacts with and promotes ubiquitination and proteasomal degradation of Sin3B (Kong et al., 2010). Our previous studies find that Rnf220 works as a novel modulator of sonic hedgehog (Shh)/Gli signaling gradient and is a key regulator of the patterning of the ventral spinal neural tube in mice (Ma et al., 2019; Ma et al., 2020). Furthermore, the development of noradrenergic neurons in locus coeruleus of the pons requires Rnf220/Zc4h2-mediated monoubiquitylation of transcription factors Phox2a and Phox2b (Song et al., 2020). However, the expression pattern of *Rnf220* in the hindbrain and its potential function in the early development of the hindbrain remain unclear.

The procedure of embryonic cells forming different tissues and organs and comprising orderly spatial structures is termed pattern formation. The DV pattern formation of the hindbrain is similar to that of the spinal cord. Along the DV axis, the progenitors in the hindbrain are divided into dp1-dp6, p0, p1, p2, pMN, and p3 domains. The post-mitotic cells generated from the dorsal neural tube are dl1-dl6, and those from the ventral neural tube are distributed into V0, V1, V2, sMNs (somatic motor neurons), and V3 domains in the mantle zone from dorsal to ventral (Lebel et al., 2007; Le Dréau and Martí, 2012; Carcagno et al., 2014).

Unlike the spinal neural tube, the neurons generated from the p3 domain of the hindbrain are the visceral motor neurons (vMNs) and 5-HTNs instead of glutamatergic neurons (Carcagno et al., 2014), and this made us interested in the development of 5-HTNs in the absence of Rnf220. During embryonic development, 5-HTNs are generated from E10.5, and vMNs are generated before E10.5 (Ding et al., 2003; Pattyn et al., 2003). In addition, lineage-tracing experiments show that the progenitors from the Olig2^+^ pMN domain also give rise to OPCs and then generate oligodendrocytes at later embryonic stages (Masahira et al., 2006). Mature oligodendrocytes express myelin basic protein (Mbp) and proteolipid protein 1 (Plp1) and are critical for the myelination of axons and involved in neurodegenerative diseases in brain (Boggs, 2006; Simons and Nave, 2015; Berry et al., 2020).

The Shh signal is required for the ventral neural tube patterning along the entire neural tube, but different phenotypes are reported in the mouse hindbrain compared with the spinal cord when Shh signaling was interfered as shown by the fact that the Olig2^+^ domain is expanded ventrally in the spinal cord but disappeared in the hindbrain of Gli2^−/−^ mice (Lebel et al., 2007). In this study, we examine the expression of *Rnf220* in the ventral neural tube of the hindbrain and explore the territory of different progenitor domains by the examination of the domain-specific gene expression in *Rnf220* ^Nestin^ CKO mice. Overall, there is an expansion of the dorsal and ventral domains at the expense of the middle domains in the ventral neural tube with an unchanged range (territory) of the ventral tube. Our work also establishs clear roles of Rnf220 in the development of 5-HTNs and OPCs in the hindbrain.

## Materials and Methods

### Animals, Staging, and Genotyping

All mice were maintained and handled according to guidelines approved by the Animal Committee of Tongji University School of Medicine, Shanghai, China. All mice were maintained on a C57BL/6 background. Analysis was performed only after lines were crossed to C57BL/6 for at least three generations.

The stage of mouse embryos was determined by taking the morning when the copulation plug was seen as E0.5. All genotypes described were confirmed by PCR. *Rnf220* alleles were genotyped using genome DNA prepared from tail tips. PCR primers were used as described in a previous report (Ma et al., 2019). PCR amplified DNA was analyzed on 1.5% TAE agarose gel.

**Key Resources Table:** 

Reagent or Resource	Source	Identifier
Antibodies and Dilution		
Goat anti-5-HT (1:300)	Immunostar	Cat #20079
Mouse anti-Nkx2.2 (1:20)	Developmental Studies Hybridoma Bank	Cat #74.5A5
Mouse anti-Nkx6.1 (1:40)	Developmental Studies Hybridoma Bank	Cat #F55A10
Rabbit anti-Cleaved Caspase-3 (1:300)	CST	Cat #9661S
Rabbit anti-Olig2 (1:300)	Abcam	Cat #ab109186
Rat anti-BrdU (1:1000)	Accrate Chemical	Cat #OBT0030G
Experimental Models: organisms/strains		
Mouse: *Rnf220* ^flox/flox^	Bingyu Mao’s Lab	N/A
Mouse: *Nestin*-Cre	The Jackson Laboratory	003771
Software and Algorithms		
Photoshop 2018 CC	Adobe	N/A
ImageJ	Freeware	N/A
Prism5	Graphpad	N/A

### *In situ* Hybridization Assays

Embryos were fixed in 4% paraformaldehyde (PFA) in phosphate-buffered saline (PBS, pH 7.4) for 24 h and cryoprotected with 30% sucrose in PBS. Then, 20-μm-thick transverse sections were cut on a cryostat (Leica), and *in situ* hybridization was performed as described previously (Song et al., 2011). Briefly, RNA probes for detecting *Pax3, Axin2, Msx1, Phox2b,* serotonin transporter (*Sert*)*,* tryptophan 5-hydroxylase 2 (*Tph2*), vesicular monoamine transporter 2 (*Vmat2*), monoamine oxidase A (*MaoA*), Sex determining region Y-box 10 (*Sox10*), *Mbp* and *Plp1* were generated according to the description on the website of Allen Brain Atlas (http://portal.brain-map.org/). Probes against *Rnf220*, *Shh*, *Pax6*, *Dbx1*, *Dbx2*, *Hb9*, *Chx10*, *En1,* and *Evx1* were used as described previously (Ma et al., 2019). All probes were cloned into pGEM-T vector (Promega) and transcribed by T7 or SP6 *in vitro* transcription kit (Ambion). Sections were observed and images were captured using epifluorescence microscope (80i; Nikon).

### Immunohistochemistry Analysis

For immunostaining, 20-μm-thick transverse sections were used, and the procedure is described in our previous report (Song et al., 2011). Antibodies used are listed in the Key Resources table. For double labeling using immunostaining and *in situ* hybridization, sections underwent the *in situ* hybridization procedure first. After visualization for mRNA, sections were incubated with primary antibody at 4°C overnight, followed by appropriate secondary antibody for 3 h. The sections were then processed using a Vectastain Elite ABC kit (Vector Laboratories) for 1 h, and immunoreactivity was visualized by incubation with diaminobenzidine (DAB) and H_2_0_2_.

### Bromodeoxyuridine (BrdU) Labeling

For BrdU pulse labeling experiments to analyze cell proliferation, pregnant mice received a single injection of BrdU at 50 mg/kg body weight and were sacrificed 1 h later. Sections were immersed in 0.01 M citrate buffer at 95°C for 20 min, 0.5 M HCl at 55°C for 10 min, and then washed in PBS. Treated sections were immunostained with anti-BrdU antibody as described above.

### Quantification and Statistical Analysis

To quantify the sizes of progenitor domains (*Shh* ^+^ for floor plate, Nkx2.2^+^ for p3 domain, Olig2^+^ for pMN domain, Nkx6.1^+^ region located dorsal to Olig2^+^ for p2 domain, the gap between *Dbx1* ^+^ and Nkx6.1^+^ for p1 domain, *Dbx1* ^+^ for p0 domain and *Pax3* ^+^ for dp1-dp6 domain) and the numbers of the ventral post-mitotic neurons (5-HT^+^, *Sert* ^+^, *Phox2b* ^+^, *Hb9* ^+^, *Chx10* ^+^, *En1* ^+^, and *Evx1* ^+^) and OPCs (*Sox10* ^+^), serial sections of the E10.5 or E12.5 hindbrain neural tubes were immunostained with the indicated antibodies or processed with antisense RNA probes. At least three sections around the rhombomere 5 (r5) level determined by the presence of otic vesicles in transverse sections or at indicated hindbrain level were included from each embryo. The size of each domain in images was measured using ImageJ with the Segmented Line Tool, and the percentage of each domain to the whole DV extent of the VZ was calculated. The number of post-mitotic cells indicated with different markers and BrdU^+^ and Cleaved Caspase-3^+^ cells were counted using the Multi-point Tool of ImageJ. Data are expressed by mean ± SEM, and two-tailed Student’s *t*-test was used for each comparison. GraphPad software was used for statistical analysis. *p*-values less than 0.05 were considered statistically significant.

## Results

### *Rnf220* is Expressed in the Ventral Half of the Hindbrain Neural Tube

**FIGURE 1:**
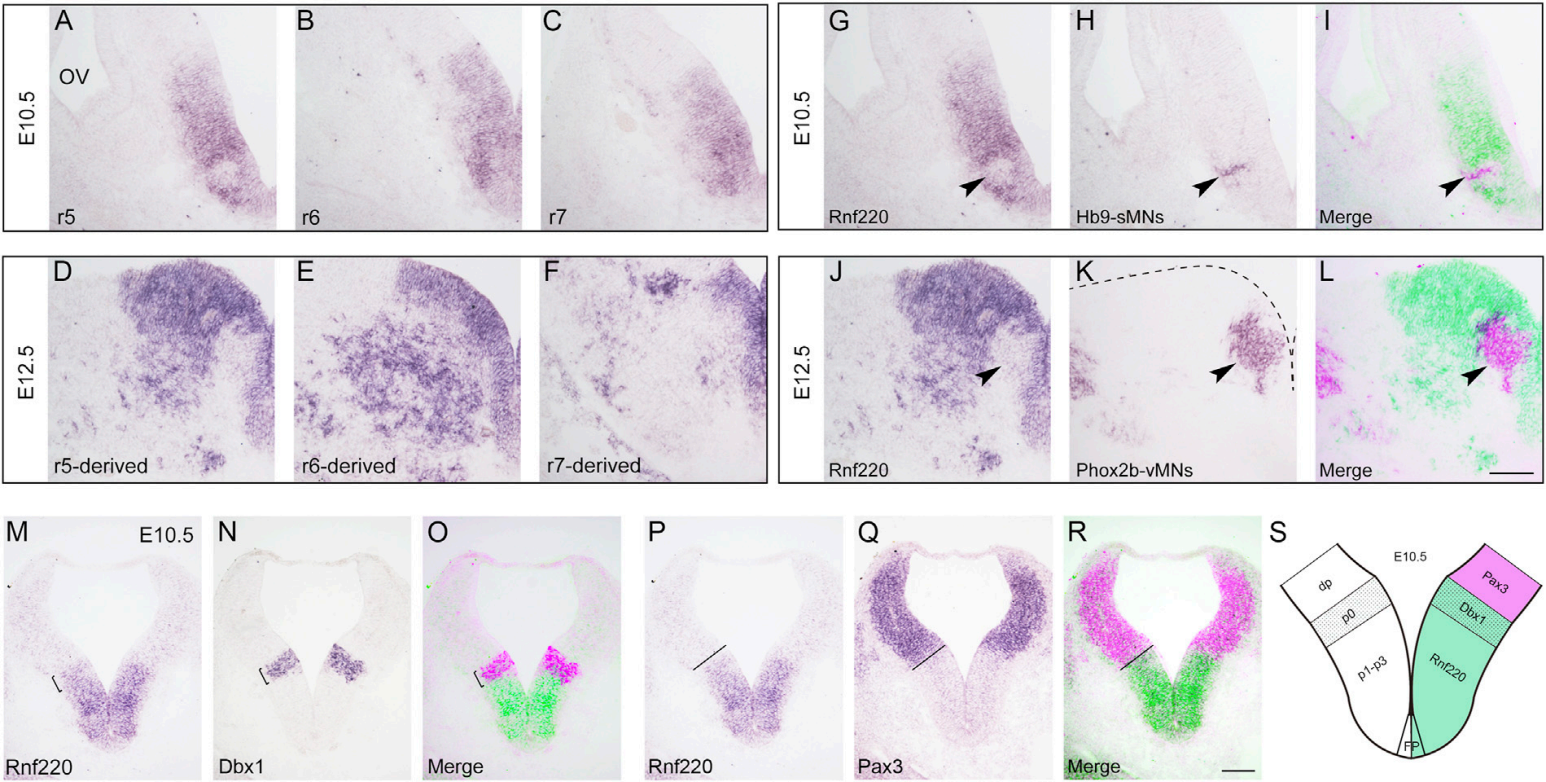
Expression of *Rnf220* in the Developing Hindbrain. **(A–C)** *In situ* hybridization for *Rnf220* of wild-type embryos at E10.5. *Rnf220* is expressed in the VZ of the ventral neural tube and a few post-mitotic cells in the mantle zone. OV, otic vesicle; r5-r7, section at rhombomere 5-7 levels. **(D–F)** *In situ* hybridization for *Rnf220* of wild-type embryos at E12.5. *Rnf220* is expressed in the VZ of the ventral neural tube and many post-mitotic cells outside the VZ. **(G–I)** *In situ* hybridization for *Rnf220* and *Hb9* on adjacent sections at the r5 level of wild-type embryos at E10.5. *Rnf220* (green) and *Hb9* (magenta) are shown by pseudocolor **(I)**. Few or very weak *Rnf220* expression is present in *Hb9* ^+^ sMNs (arrowheads). sMNs, somatic motor neurons. **(J–L)** *In situ* hybridization for *Rnf220* and *Phox2b* on adjacent sections at the r5-derived level of wild-type embryos at E12.5. *Rnf220* (green) and *Phox2b* (magenta) are shown by pseudocolor **(L)**. *Rnf220* is not expressed in *Phox2b* ^+^ vMNs (arrowheads). Dashed line in **(K)** shows the boundary of the hindbrain. vMNs, visceral motor neurons. Scale bar, 100 μm **(L)**; also applies to **A–K)**. **(M–O)** *In situ* hybridization for *Rnf220* and *Dbx1* (a p0 domain marker) on adjacent slides of E10.5 wild-type embryos. *Dbx1* (magenta) and *Rnf220* (green) are shown by pseudocolor **(O)**. The uppermost territory of *Rnf220* expression domain corresponds well with that of *Dbx1* (brackets). **(P–R)** *In situ* hybridization for *Rnf220* and *Pax3* (a dp1-dp6 domain marker) on adjacent slides of E10.5 wild-type embryos. The expression domain of *Rnf220* is adjacent to that of *Pax3* (dashed lines). *Pax3* (magenta) and *Rnf220* (green) are shown by pseudocolor (R). Scale bar, 100 μm **(R)**; also applies to **(M–Q)**. **(S)** Diagram showing the expression pattern of *Rnf220* in the E10.5 hindbrain. *Rnf220* is expressed from p0 to p3 domain and in the floor plate **(FP)**, floor plate.

Our previous study reports that *Rnf220* is expressed in the ventral half of the spinal neural tube (Ma et al., 2019), and we assumed that it is also the case for the hindbrain. To test this, we carried out *in situ* hybridization in wild-type embryos at E10.5 and E12.5. As expected, intense expression of *Rnf220* was present in the VZ of the ventral neural tube and weak expression was observed in the floor plate at E10.5 (Figures 1A–C). In the E12.5 hindbrain, *Rnf220* expression was also localized in many post-mitotic cells in the ventral mantle zone (Figures 1D–F). However, in the VZ region at the level of r5, where *Hb9* ^+^ sMNs are generated, much less *Rnf220* expression was present at E10.5 (Figures 1G–I), and the corresponding VZ region containing *Phox2b* ^+^ vMNs at E12.5 had weak, if any, *Rnf220* transcripts (Figures 1J–L). To further localize its distribution pattern, we performed *in situ* hybridization for *Rnf220* and *Dbx1*, a p0 domain marker (Pierani et al., 2001), and also for *Pax3*, a dp1-dp6 domain marker (Moore et al., 2013), on adjacent sections at E10.5. The result shows that the uppermost territory of *Rnf220* expression domain corresponds well with that of *Dbx1* (Figures 1M–O, S) and is adjacent to that of *Pax3* (Figures 1P–R, S), confirming that *Rnf220* is restrictively expressed in the ventral half of the hindbrain neural tube, covering ventral p0-p2, pMN, p3 domains, and the floor plate (Figure 1S).

### Progenitor Domains are Altered in the Ventral Hindbrain Neural Tube of *Rnf220* ^Nestin^ CKO Embryos

To investigate the function of Rnf220 in the early development of the hindbrain, we crossed *Rnf220* ^flox/flox^ (Ma et al., 2019) with a *Nestin*-Cre line (Tronche et al., 1999), which expresses Cre recombinase in the neuronal and glial cell precursors from E10.5. *Nestin*-Cre:*Rnf220* ^flox/+^ mice were crossed with *Rnf220* ^flox/flox^ or *Rnf220* ^flox/+^ mice, and the resulting embryos were genotyped using PCR and processed for phenotypic analysis. *Rnf220* ^Nestin^ CKO (*Nestin*-Cre:*Rnf220* ^flox/flox^) embryos were obtained, and littermates with other genotypes showing no detectable phenotypes were used as controls. The *Rnf220* ^Nestin^ CKO embryos were found to be neonatal lethal, and the deletion of *Rnf220* expression in these embryos was confirmed by *in situ* hybridization using an exon2-specific probe (Supplementary Figure S1). Residual weak *Rnf220* expression in the ventral-most hindbrain is likely caused by incomplete or absent Cre activity in these cells.

**FIGURE 2:**
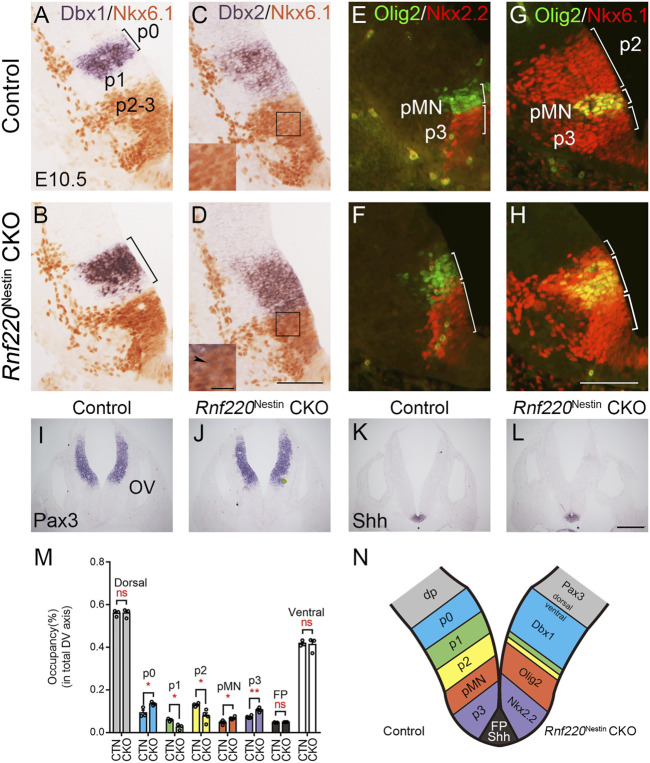
The Progenitor Domains are Altered in the Developing Ventral Hindbrain of *Rnf220* ^Nestin^ CKO Embryos at E10.5. **(A,B)** *In situ* hybridization for *Dbx1* and immunostaining for Nkx6.1 show that *Dbx1* expression is expanded ventrally to the dorsal boundary of the Nkx6.1^+^ domain in *Rnf220* ^Nestin^ CKO embryos **(B)**, whereas there is a gap region (i.e., the p1 domain) between the Nkx6.1^+^ and Dbx1^+^ domains in control embryos **(A)**. **(C,D)** *In situ* hybridization for *Dbx2* and immunostaining for Nkx6.1 show that *Dbx2* expression is expanded ventrally into the Nkx6.1^+^ domain in *Rnf220* ^Nestin^ CKO embryos **(D)** (arrowhead), whereas a clear boundary between the Nkx6.1^+^ and Dbx2^+^ domains is present in the control neural tubes **(C)**. Scale bar, 100 μm **(D)**; also applies to **(A–C)**. Insets are high-magnification images of boxed areas in **(C,D)**. Scale bar, 25 μm. **(E,F)** Double immunostaining shows that both the Nkx2.2^+^ (red) p3 domain and the Olig2^+^ (green) pMN domain are expanded along the DV axis of the VZ in *Rnf220* ^Nestin^ CKO embryos **(F)** relative to controls **(E)**. **(G,H)** Double immunostaining for Nkx6.1 (red) and Olig2 (green) shows a dorsal shift of the Nkx6.1^+^/Olig2^+^ pMN domain and a reduction of the p2 domain in the *Rnf220* ^Nestin^ CKO neural tubes **(H)** compared with controls **(G)**. The p2 domain corresponds to the Nkx6.1^+^ region located dorsal to the Nkx6.1^+^/Olig2^+^ pMN domain. Scale bar, 100 μm **(H)**; also applies to **(E-G)**. **(I,J)** *In situ* hybridization for *Pax3* of control **(I)** and CKO embryos **(J)** at E10.5. OV, otic vesicle. **(K,L)** *In situ* hybridization for *Shh* of control **(K)** and CKO embryos **(L)** at E10.5. Scale bar, 100 μm **(L)**; also applies to **(I–K)**. **(M)** Quantification of the sizes of each progenitor domain in the neural tube of control and *Rnf220* ^Nestin^ CKO embryos. The size is shown by its proportion in the whole extent of the VZ along the DV axis. CTN, control; CKO, *Rnf220* ^Nestin^ CKO. Dorsal, Pax3^+^ domain; p0, Dbx1^+^ domain; p1, gap region between the Dbx1^+^ and Nkx6.1^+^ domains; p2, Olig2^-^ domain in the dorsal part of the Nkx6.1^+^ region; pMN, Olig2^+^ domain; p3, Nkx2.2^+^ domain; FP, floor plate, Shh^+^ domain. Data are expressed by mean ± SEM. Student’s *t*-test (*n*≥3 for each). *, *p*<0.05; **, *p*<0.01; ns, no significant difference. **(N)** Diagram showing the changes of the progenitor domains of the ventral hindbrain in *Rnf220* ^Nestin^ CKO embryos at E10.5. The loss of *Rnf220* leads to the expansion of the Dbx1^+^ p0, Olig2^+^ pMN, and Nkx2.2^+^ p3 domains in the ventral hindbrain neural tube at the expense of the middle domains (i.e., the p1 and p2 domains) without affecting the extent of the dorsal neural tube.

A group of genes was used to determine whether the deletion of *Rnf220* affects the progenitor domains in the ventral hindbrain neural tube at E10.5. *In situ* hybridization for *Dbx1* and immunostaining for Nkx6.1 (a marker for p2-pMN and p3 domains) showed that the p0 domain was expanded ventrally at the expense of the p1 domain (a gap between the Nkx6.1^+^ and Dbx1^+^ domains) in *Rnf22*0^Nestin^ CKO embryos (Figures 2A,B, M,N). In addition, the Dbx2^+^ p0-p1 and Nkx6.1^+^ domains were located next to each other without overlapping in control neural tubes (Figure 2C). In contrast, there was an increase of *Dbx2* expression in the p0 and p1 domains and weak *Dbx2* expression was expanded ventrally into the Nkx6.1^+^ domain in *Rnf220* ^Nestin^ CKO embryos (Figure 2D). Double immunostaining for Olig2 (a pMN domain marker) and Nkx2.2 (a p3 domain marker) showed that both the p3 and pMN domains were dorsally expanded in the CKO embryos compared with controls (Figures 2E,F, M,N). The increase of the pMN and p3 domains were further confirmed by double immunostaining for Olig2 and Nkx6.1 (Figures 2G,H). The reduction of p2 domain was confirmed as shown by the Nkx6.1^+^ region located dorsal to the Olig2^+^/Nkx6.1^+^ pMN domain (Figures 2G,H, M,N), and the double staining also provided evidence showing the pMN domain was shifted dorsally (Figures 2G,H, N). Note that the extent of the ventral neural tube from the p0 domain to the floor plate was not changed (Figures 2M,N).

We next moved to examine if the floor plate and dorsal neural tube was affected in *Rnf220* ^Nestin^ CKO embryos. The floor plate shown by the expression of *Shh* and the dorsal neural tube shown by *Pax3* were not obviously changed (Figures 2I–L). Taken together, we conclude that the loss of *Rnf220* led to the expansions of the dorsal-most (p0) and ventral-most (pMN and p3) domains at the expense of the middle domains (i.e., the p1 and p2 domains) in the ventral hindbrain neural tube (Figure 2N).

### No Obvious Changes in Proliferation and Cell Death in the Developing Ventral Hindbrain of *Rnf220* ^Nestin^ CKO Embryos at E10.5

**FIGURE 3:**
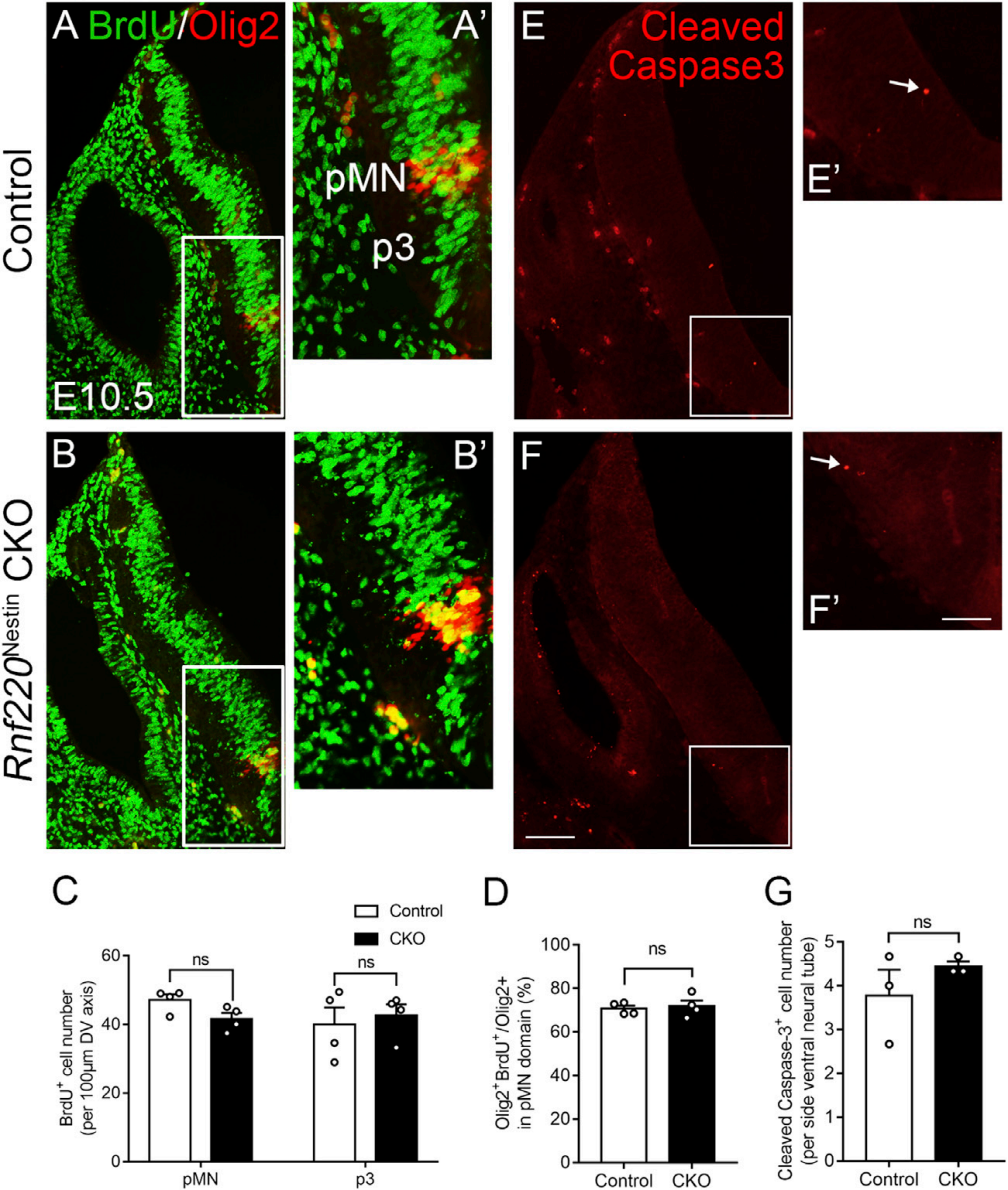
No Significant Changes in Proliferation and Cell Death in the Ventral Hindbrain of *Rnf220* ^Nestin^ CKO Embryos at E10.5. **(A–B)** Double immunostaining for BrdU (green) and Olig2 (red) shows no obvious changes of proliferation in the ventral VZ of the hindbrain between *Rnf220* ^Nestin^ CKO embryos and controls. **(A’,B’)** are high-magnification images of boxed areas in **(A,B)**, respectively. **(C)** Quantification of the numbers of BrdU^+^ cells per 100 μm-length in the Olig2^+^ pMN domain and p3 domain of control and *Rnf220* ^Nesin^ CKO embryos at E10.5. The p3 domain corresponds to the BrdU^+^ region located ventral to the Olig2^+^ pMN domain. ns, no significant difference. **(D)** Quantification of the percentages of BrdU/Olig2 double-labeled cells in Olig2^+^ cells in the pMN domain of control and *Rnf220* ^Nesin^ CKO embryos at E10.5. **(E,F)** Immunostaining for Cleaved Caspase-3 shows similar cell death in *Rnf220* ^Nestin^ CKO embryos compared with controls. **(E’,F’)** are high-magnification images of boxed areas in **(E,F)**, respectively. Scale bar, 100 μm **(F)**; also applies to **(A,B,E)**; 50 μm **(F’)**; also applies to **(A’,B’,E’)**. **(G)** Quantification of the numbers of Cleaved Caspase-3^+^ cells in the ventral neural tube in the two groups at E10.5. Data are expressed by mean ± SEM. Student’s *t*-test (n ≥ 3 for each).

The loss of *Rnf220* resulted in alterations of progenitor domains of the ventral hindbrain neural tube. We wondered if the changes in progenitor domains were due to abnormal proliferation or cell death. To explore such a possibility, we performed pulse labeling of BrdU and immunostaining of Cleaved Caspase-3 to label cell proliferation and cell death, respectively. Overall, BrdU labeling was not obviously different between the two groups (Figures 3A,B). To clearly define the territory of the pMN and p3 domains for quantification of BrdU^+^ cells, double staining for BrdU and Olig2 was carried out. Because the DV extents of the pMN and p3 domains were increased, we compared the numbers of BrdU^+^ cells per 100 μm-length in the two domains and found no significant difference between *Rnf220* ^Nestin^ CKO and control embryos (Figure 3C). Next, we compared percentages of BrdU/Olig2 double-labeled cells in Olig2^+^ cells in the pMN domain and revealed no significant difference either (Figure 3D). In addition, no significant difference was detected in the numbers of Cleaved Caspase-3^+^ cells between the two groups (Figures 3E–G). However, proliferation and cell death were altered at E12.5. *Rnf220* ^Nestin^ CKO embryos showed lower density of BrdU^+^ cells in the ventral VZ than control embryos (Supplementary Figures S2A–C), and more Cleaved Caspase-3^+^ cells were detected in the ventral hindbrain of CKO embryos (Supplementary Figures S2D–F). These data suggest that the changes of progenitor domains in *Rnf220* ^Nestin^ CKO embryos at E10.5 are not due to abnormal proliferation or cell death, but it remains to be established how *Rnf220* mutation affects neural stem cell proliferation and cell death at later stages.

### Alterations of Post-mitotic Neurons in the Developing Ventral Hindbrain of *Rnf220* ^Nestin^ CKO Embryos

**FIGURE 4:**
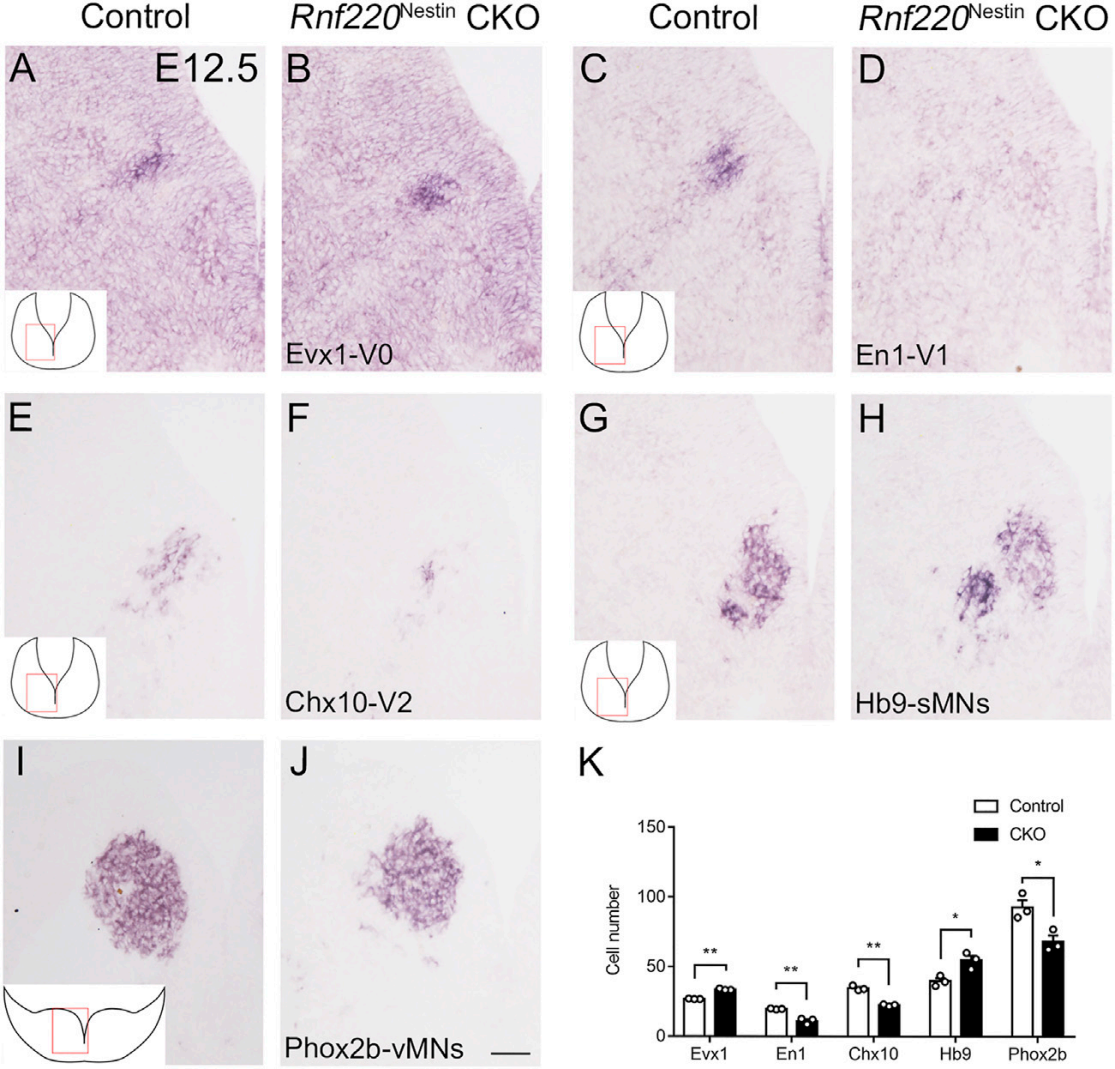
Changes in the Domains of Post-mitotic Neurons in the Ventral Hindbrain Neural Tube of *Rnf220* ^Nestin^ CKO Embryos at E12.5. **(A,B)** More *Evx1* ^+^ V0 neurons are observed in *Rnf220* ^Nestin^ CKO embryos **(B)** than in controls **(A)**. **(C,D)** The *En1* ^+^ V1 neurons are hardly detected in *Rnf220* ^Nestin^ CKO embryos **(D)** compared with the V1 neuronal domain in controls **(C)**. **(E,F)** The number of *Chx10* ^+^ V2 neurons is reduced in *Rnf220* ^Nestin^ CKO embryos **(F)** compared with controls **(E)**. **(G,H)** *Hb9* ^+^ somatic motor neurons (sMNs) are increased in *Rnf220* ^Nestin^ CKO embryos **(H)** compared with controls **(G)**. **(I,J)** *Phox2b* ^+^ visceral motor neurons (vMNs) are reduced in *Rnf220* ^Nestin^ CKO embryos **(J)** compared with controls **(I)**. Scale bar, 50 μm **(J)**; also applies to **(A–I)**. Insets **(A,C,E,G,I)** are schematic diagrams of the hindbrain level and position shown in **(A–J)**. **(K)** Quantification of different groups of post-mitotic neurons in the ventral hindbrain neural tube of control and *Rnf220* ^Nestin^ CKO embryos. Data are expressed by mean ± SEM. Student’s *t*-test (*n*=3 for each). *, *p*<0.05; **, *p*<0.01.

Having observed dramatic changes in the progenitor domains of the ventral hindbrain neural tube, it is likely that their progeny may also be altered in the absence of *Rnf220*. The progenitor domains that express *Rnf220* within the ventral hindbrain neural tube of control embryos include p0, p1, p2, pMN, and p3 domains, which give rise to V0, V1, V2 interneurons; sMNs; OPCs; vMNs; and 5-HTNs, respectively. As expected, more post-mitotic neurons from the p0 domain were observed as shown by the increased expression of *Evx1* in *Rnf220* ^Nestin^ CKO embryos relative to its expression in the control embryos at E12.5 (Figures 4A,B,K). More ventrally, the *En1* ^+^ V1 and *Chx10* ^+^ V2 interneurons were markedly reduced or abolished in CKO embryos (Figures 4C–F,K). The sMNs from the pMN domain express *Hb9*, which were significantly increased in *Rnf220* ^Nestin^ CKO embryos (Figures 4G,H,K). Unexpectedly, *Phox2b* ^+^ vMNs from the p3 domain in *Rnf220* ^Nestin^ CKO embryos were slightly reduced compared with those in the control group (Figures 4I–K) although the p3 domain was increased in *Rnf220* ^Nestin^ CKO embryos (Figures 2E–H, M,N). In addition, there was ectopic distribution of *Hb9* ^*+*^ cells at the r6-derived level of the hindbrain in *Rnf220* ^Nestin^ CKO embryos, whereas they were only present at the r5- and r7-derived hindbrain in control mice (Supplementary Figure S3). This is perhaps due to the migration defect of r5-derived neurons of the abducens nerve and r7-derived neurons associated with the hypoglossal nerve, but further experiment is needed. Thus, the deletion of *Rnf220* not only changed the progenitor domains, but also altered their progeny in the ventral hindbrain correspondingly.

### 5-HTNs are Increased in *Rnf220* ^Nestin^ CKO Mice

**FIGURE 5:**
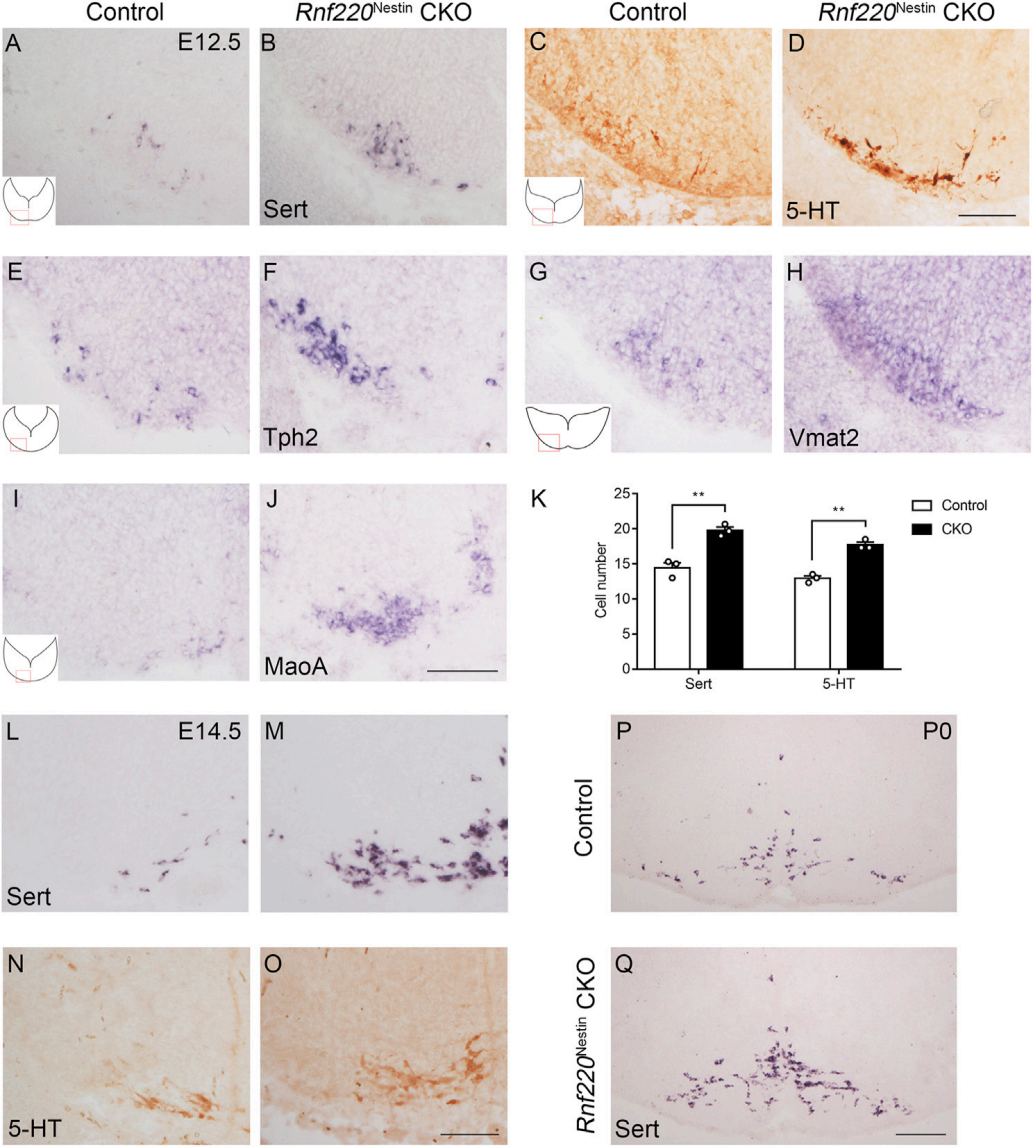
The Number of 5-HTNs is Increased in the Hindbrain of *Rnf220* ^Nestin^ CKO Mice. **(A,B)** *In situ* hybridization for *Sert* shows increased number of 5-HTNs in *Rnf220* ^Nestin^ CKO embryos **(B)** compared with controls **(A)** at E12.5. **(C,D)** DAB staining for 5-HT shows more 5-HTNs in *Rnf220* ^Nestin^ CKO embryos **(D)** than in controls **(C)** at E12.5. Scale bar, 100 μm **(D)**; also applies to **(A–C)**. **(E–J)** *In situ* hybridization for *Tph2*, *Vmat2* and *MaoA* shows increased expressions of these genes in *Rnf220* ^Nestin^ CKO embryos **(F,H,J)** compared with controls **(E,G,I)**. Scale bar, 50 μm **(J)**; also applies to **(E–I)**. Insets **(A,C,E,G,I)** are schematic diagrams of the hindbrain level and position shown in **(A–J)**. **(K)** Quantification of *Sert* ^+^ and 5-HT^+^ neurons. Data are expressed by mean ± SEM. Student’s *t*-test (*n*=3 for each). **, *p*<0.01. **(L,M)** *In situ* hybridization for *Sert* shows increased number of 5-HTNs in *Rnf220* ^Nestin^ CKO embryos **(M)** compared with controls **(L)** at E14.5. **(N,O)** DAB staining for 5-HT shows more 5-HTNs in *Rnf220* ^Nestin^ CKO embryos **(O)** than in controls **(N)** at E14.5. Scale bar, 100 μm **(O)**; also applies to **(L–N)**. **(P,Q)** *In situ* hybridization for *Sert* shows more 5-HTNs in *Rnf220* ^Nestin^ CKO mice **(Q)** than in controls **(P)** at postnatal day 0 (P0). Scale bar, 200 μm **(Q)**; also applies to **(P)**.

Neurons that are capable of synthesis and release the essential neurotransmitter 5-HT are called 5-HTNs. 5-HT is synthesized by Tph2 (Walther et al., 2003) and aromatic L-amino-acid decarboxylase (Aadc) (Albert et al., 1987) from tryptophan. Then, 5-HT is packaged into synaptic vesicles by Vmat2 (Weihe et al., 1994). Extracellular 5-HT can be transported back into the cells by Sert (Hoffman et al., 1991). In the cells, 5-HT is broken down by MaoA and MaoB (Luque et al., 1995). As 5-HTNs are generated from the p3 domain, which was increased in *Rnf220* ^Nestin^ CKO embryos, we examined whether the development of 5-HTNs was affected by knocking out *Rnf220*. *In situ* hybridization for *Sert* and immunostaining for 5-HT showed that the number of 5-HTNs was increased in *Rnf220* ^Nestin^ CKO embryos at E12.5 (Figures 5A–D,K). To further confirm this, *in situ* hybridization for *Tph2*, *Vmat2*, and *MaoA* were performed (Figures 5E–J), and expressions of all these genes were increased. We also detected the numbers of 5-HTNs at E14.5 (Figures 5L–O) and postnatal day 0 (Figures 5P,Q), which were still increased in *Rnf220* ^Nestin^ CKO mice.

### OPCs are also Increased in *Rnf220* ^Nestin^ CKO Embryos

**FIGURE 6:**
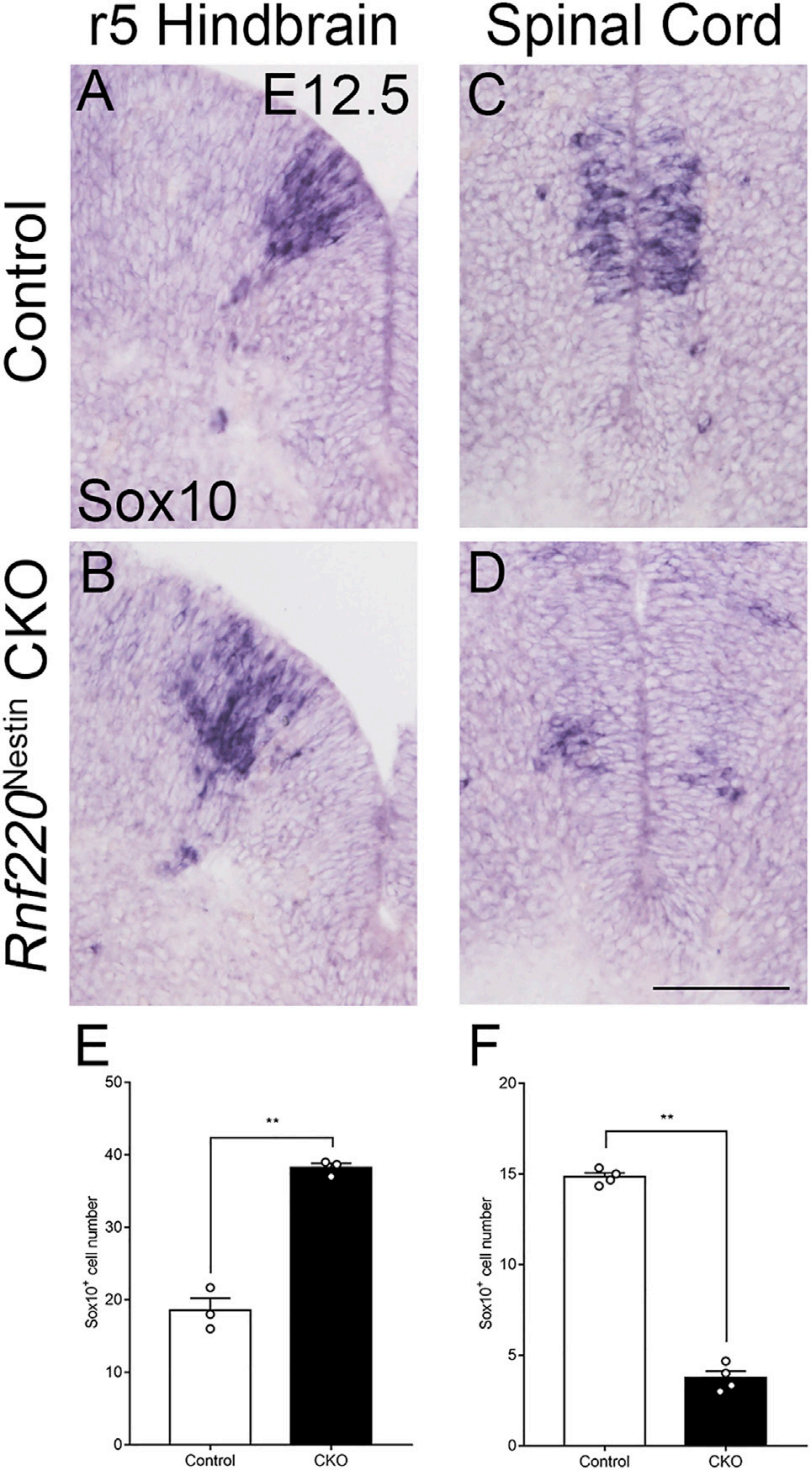
OPCs are Increased in the Hindbrain but not the Spinal Cord of *Rnf220* ^Nestin^ CKO Embryos. **(A–D)** *In situ* hybridization for *Sox10* at E12.5. At the r5-derived level of the hindbrain, the number of the OPCs are increased in *Rnf220* ^Nestin^ CKO embryos **(B)** compared with controls **(A)**, whereas the number is decreased in CKO **(D)** compared with that of the control **(C)** in the spinal cord. Scale bar, 100 μm **(D)**; also applies to **(A–C)**. **(E)** Quantification of *Sox10* ^+^ cells of the r5 hindbrain at E12.5. **(F)** Quantification of Sox10^+^ cells of the spinal cord at E12.5. Data are expressed by mean ± SEM. Student’s *t*-test (n ≥ 3 for each). **, *p*<0.01.

OPCs are generated in the ventral hindbrain at E12.5 from the Olig2^+^ pMN domain (Richardson et al., 2006), which was shifted and expanded dorsally in *Rnf220* ^Nestin^ CKO embryos as mentioned above. This prompted us to examine if the generation of OPCs was altered in the CKO embryos. We observed that *Sox10* ^+^ OPCs in the ventral hindbrain were increased and shifted dorsally at E12.5 (Figures 6A,B,E). As development progressed, mature oligodendrocytes, marked by *Mbp* and *Plp1*, were located in a narrow region in the mantle zone along the VZ in control embryos, whereas they displayed a dispersed distribution pattern in the *Rnf220* ^Nestin^ CKO hindbrain at E14.5 (Supplementary Figure S4). In addition, OPCs are also generated in the ventral spinal cord, but its development in Rnf220-deficient mice has not been examined. OPCs in the spinal cord, however, were diminished in CKO embryos at E12.5 (Figures 6C,D,F). Note that the reduction coincides with the decrease of the Olig2^+^ domain of the *Rnf220* ^−/−^ spinal neural tube (Ma et al., 2019). Thus, there is a different phenotype in the ventral neural tube patterning in the absence of *Rnf220*: An increased Olig2^+^ domain led to an increase of OPCs in the hindbrain, but a reduced Olig2^+^ domain resulted in a decrease of OPCs in the spinal cord.

## Discussion

**FIGURE 7:**
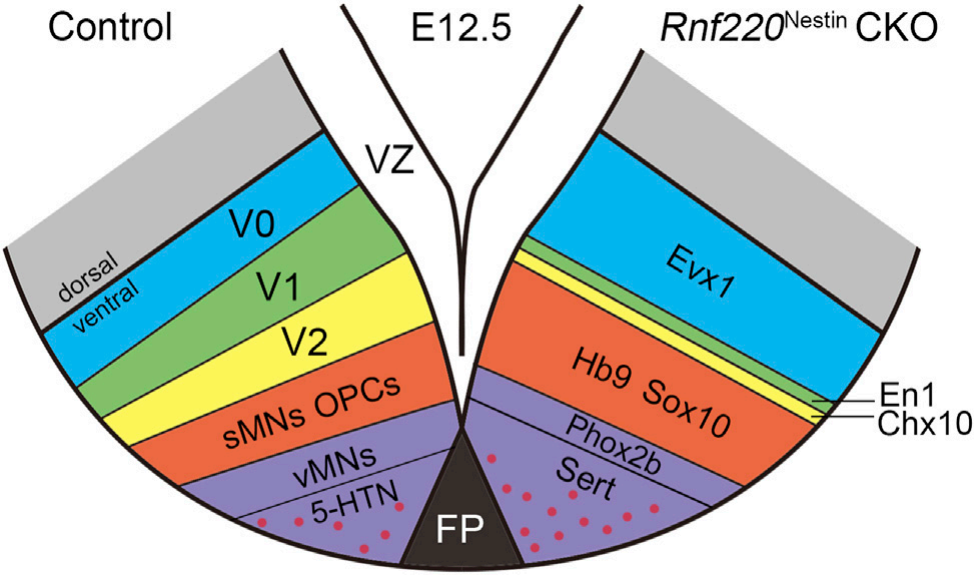
Diagram Showing the Changes of Post-mitotic Cells in the Ventral Hindbrain of *Rnf220* ^Nestin^ CKO Embryos at E12.5. The loss of *Rnf220* leads to the expansion of the Evx1^+^ V0, Hb9^+^ sMNs and Sox10^+^ OPCs domains, whereas the En1^+^ V1 and Chx10^+^ V2 domains are reduced. Within the domain differentiated from p3, the Phox2b^+^ vMNs domain decreases slightly but the number of 5-HTNs is increased.

Our and other previous studies show that *Rnf220* is expressed in the ventral spinal neural tube (Kim et al., 2018; Ma et al., 2019). The present study shows that this restricted expression pattern is also present in the hindbrain neural tube with the exception of *Hb9* ^+^ sMNs and *Phox2b* ^+^ vMNs, where few or weak *Rnf220* transcripts are distributed (Figure 1). Consistent with the expression pattern, our data from *Rnf220* ^Nestin^ CKO embryos demonstrates that Rnf220 is involved in the DV patterning of the hindbrain neural tube in mice. The loss of *Rnf220* alters neural progenitor domains: the p0, pMN, and p3 domains located at the two ends of the ventral tube are increased, and the p1 and p2 domains located in the middle are decreased; their progeny show similar changes (Figures 2, 7).

Shh signaling plays crucial roles in patterning the ventral neural tube of both the spinal cord and hindbrain by controlling the expressions of several patterning genes (e.g., Olig2, Nkx2.2, and Nkx6.1) through opposing gradients of repressor and activator forms of Gli transcription factors (Ruiz i Altaba, 1998; Cohen et al., 2015; Ma et al., 2019). Our previous study shows that the deletion of *Rnf220* does not affect *Shh* expression in the notochord or the floor plate in the spinal cord but affects the functional balance between two forms of Glis via the K63-linked ubiquitination mechanism (Ma et al., 2019). The deletion of *Rnf220* in the spinal neural tube leads to the expansions of the two end domains (p0 and p3) with reductions of those in the middle (p1, p2, and pMN) and intact expression of *Shh* (Ma et al., 2019). Generally, the defective neural tube patterning in the hindbrain of *Rnf220* ^Nestin^ CKO embryos is very similar to that in the spinal neural tube, but there is a difference in the pMN domain: reduced in the spinal cord but expanded in the hindbrain. The alterations led to similar changes in the numbers of sMNs: increased in the hindbrain (Figure 4) but reduced in the spinal cord (Ma et al., 2019). Overall, *Rnf220* is homogeneously expressed in the VZ of the ventral hindbrain, but its expression is much reduced in the pMN domain at the level of generation of sMNs shown by *Hb9* and devoid in the progenitors of the visceral motor neuron domain at the level of generation of vMNs indicated by *Phox2b* (Figure 1). Both Shh and Glis are proposed to work in concentration-associated gradient fashions, and the discontinued expression of *Rnf220* in the ventral VZ may affect the shape of the gradients leading to the distinct alteration of the pMN domain in the hindbrain of *Rnf220* ^Nestin^ CKO embryos.

Although the neural tube patterning of the ventral hindbrain is altered in the absence of Rnf220, the proliferation of neural progenitors in the VZ seems not to be affected as shown by BrdU labeling at E10.5 (Figures 3A–D). However, BrdU^+^ cells are significantly reduced in the VZ of *Rnf220* ^Nestin^ CKO embryos at E12.5 (Supplementary Figures S2A–C), suggesting the proliferation capability is reduced during the embryonic development. Our previous studies show that the roles of Rnf220 in regulating neural development is achieved by controlling the stability and activity of some key genes (e.g., transcription factors) via ubiquitylation mechanisms (Ma et al., 2019; Song et al., 2020), and thus, exploring possible interactions between Rnf220 and genes involved in the proliferation of neural stem cells is required in future studies.

Different progenitor domains give rise to distinct types of neurons. Consistent with the changes in progenitor domains, more V0 interneurons and sMNs are observed, whereas V1 and V2 interneurons are significantly reduced or lost in *Rnf220* ^Nestin^ CKO embryos at later embryonic stages (Figures 4, 7). The loss of hindbrain V2 neurons might account for the neonatal death of the *Rnf220* ^Nestin^ CKO mice as the medullary V2a neurons are required for central respiratory rhythm generation in mice (Crone et al., 2012). Different from the spinal neural tube, the p3 domain in the hindbrain successively gives rise to vMNs and 5-HTNs (Pattyn et al., 2003). Interestingly, the number of vMNs is slightly reduced, and the number of 5-HTNs is significantly increased (Figures 4, 5, 7). Thus, although the p3 domain is expanded, the successively generated neurons from this domain are not equally affected in the absence of *Rnf220*. Future research is required to clarify whether the progenitor identity within the p3 domain was changed in *Rnf220* ^Nestin^ CKO embryos.

The pMN domain gives rise to sMNs first and OPCs at later embryonic stages in both the spinal and hindbrain neural tube (Zhou et al., 2000; Novitch et al., 2001; Zannino and Appel, 2009). Intriguingly, *Hb9* ^+^ sMNs and *Sox10* ^+^ OPCs are increased in the hindbrain, but they are decreased in the spinal cord (Figures 4, 6; Ma et al., 2019). Further studies are needed to explore the mechanism concerning the different effects of Rnf220 in the production of sMNs and OPCs between the hindbrain and spinal cord. It is also of interest to study if Rnf220 is implicated in the maintenance of 5-HTNs and oligodendrocytes in the adult brain as these two types of cells are involved in mental disorders and neurodegenerative diseases.

Dysfunctions of the central 5-HT system are implicated in psychiatric disorders in human and abnormal behaviors in mice (Stein and Stahl, 2000; Dai et al., 2008; Dalley and Roiser, 2012; Jia et al., 2014; Song et al., 2016; Daut and Fonken, 2019; Chen et al., 2021). Our data suggest that Rnf220 might play an important role in maintaining the homeostasis of the 5-HT system, which infers that Rnf220 might be a risk gene in mental diseases.

In summary, Rnf220 plays an indispensable role in the early development of the hindbrain, which is evidenced by drastic alterations of neural progenitor domains in the ventral hindbrain of *Rnf220* ^Nestin^ CKO mice. These results also reveal a conserved function of Rnf220 in regulating neural tube patterning in the early development of the spinal cord and hindbrain with difference in affecting the generation of post-mitotic cells such as sMNs, OPCs, and those located only in the hindbrain (i.e., 5-HTNs).

## Data Availability

The original contributions presented in the study are included in the article/Supplementary Material, further inquiries can be directed to the corresponding authors.
