# Supplementary material for: Rnf220 is Implicated in the Dorsoventral Patterning of the Hindbrain Neural Tube in Mice
Source: Front Cell Dev Biol. 2022 Mar 24;10:831365. doi: 10.3389/fcell.2022.831365 (PMC8988044; doi:10.3389/fcell.2022.831365)
Supplement: Supplementary file 1 [file DataSheet1.PDF]

Rnf220 is implicated in the dorsoventral patterning of the hindbrain neural tube in mice

Yu-Bing Wang[1], Ning-Ning Song[2,3], Lei Zhang[1], Pengcheng Ma[4], Jia-Yin Chen[2,3], Ying Huang[2,3], Ling Hu[2,3], Bingyu Mao[4,*] and Yu-Qiang Ding[1,2,3,*]

[1] Key Laboratory of Arrhythmias, Ministry of Education, East Hospital, and Department of Anatomy and Neurobiology, Tongji University School of Medicine, Shanghai 200092, China.

[2] Department of Laboratory Animal Science, Fudan University, Shanghai 200032, China.

[3] State Key Laboratory of Medical Neurobiology and MOE Frontiers Center for Brain Science, Institutes of Brain Science, Fudan University, Shanghai 200032, China.

[4] State Key Laboratory of Genetic Resources and Evolution, Kunming Institute of Zoology, Chinese Academy of Sciences, Kunming 650223, China.

[*] Correspondence to:

Bingyu Mao, mao@mail.kiz.ac.cn; Yu-Qiang Ding, dingyuqiang@vip.163.com

Supplementary Material

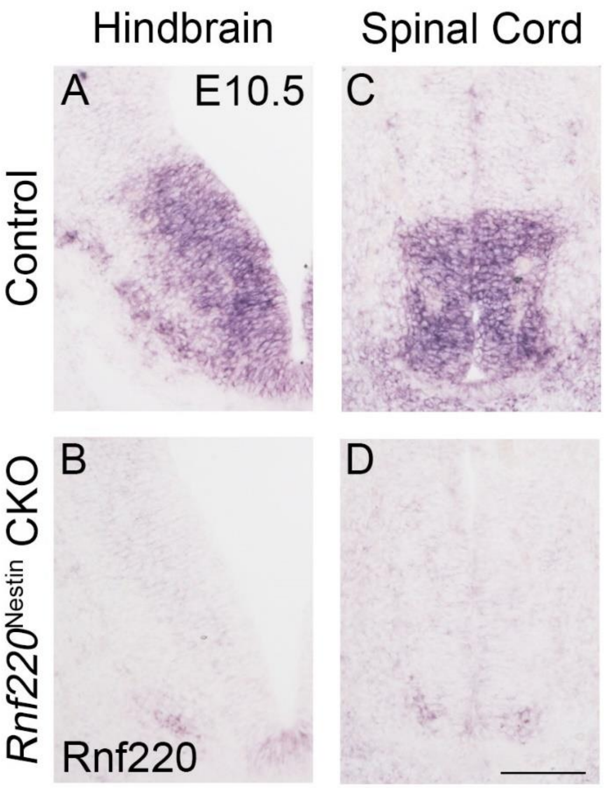

Figure S1. Deletion of *Rnf220* in CKO embryos at E10.5.

(A-D) *In situ* hybridization for *Rnf220* of control and CKO embryos at E10.5. In control embryos, *Rnf220* is expressed in the VZ and mantle zone of the ventral hindbrain (A) and spinal cord (C), while it is hardly detected in the CKO hindbrain (B) and spinal cord (D). Scale bar, 100μm (D; also applies to A-C).

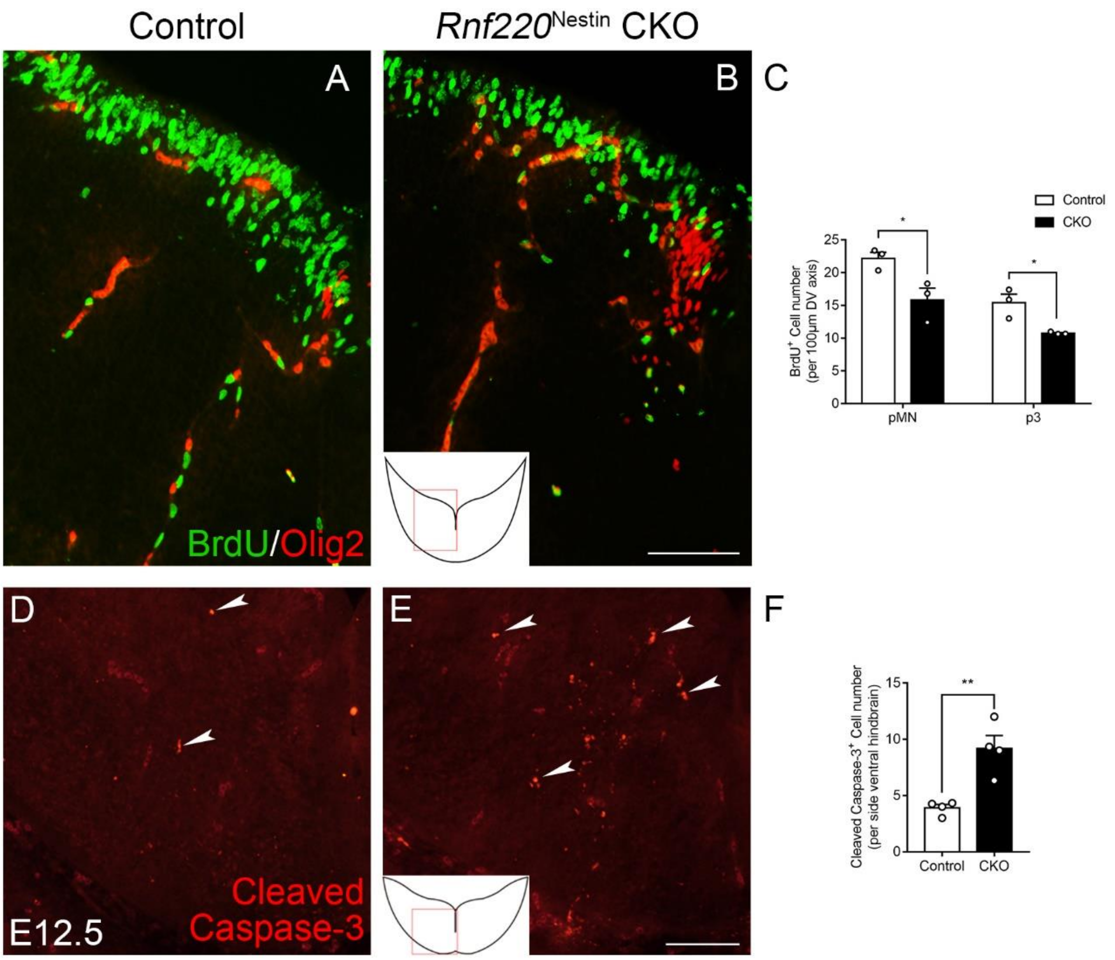

Figure S2. Cell Proliferation and cell death are altered in the ventral hindbrain of *Rnf220*^Nestin CKO embryos at E12.5.

(A, B) Double immunostaining for BrdU (green) and Olig2 (red) shows lower density of BrdU+ cells in the ventral VZ of the *Rnf220*^Nestin CKO embryos (B) compared with controls (A) at E12.5. Scale bar, 100μm (B; also applies to A).

(C) Quantification of the numbers of BrdU+ cells in the Olig2+ pMN domain and p3 domain of control and *Rnf220*^Nesin CKO embryos at E12.5. *, $p<0.05$.

(D, E) Immunostaining for Cleaved Caspase-3 shows more cell death in the ventral hindbrain of *Rnf220*^Nestin CKO embryos (E) than in controls (D, arrowheads) at E12.5. Scale bar, 100μm (E, also applies to D).

Insets (B, E) are schematic diagrams of the hindbrain level and position shown in A, B, D and E.

(F) Quantification of the numbers of Cleaved Caspase-3+ cells in the ventral neural tube in the two groups at E12.5. Data are expressed by mean ± SEM. Student's t test

(n≥3 for each). **, $p<0.01$.

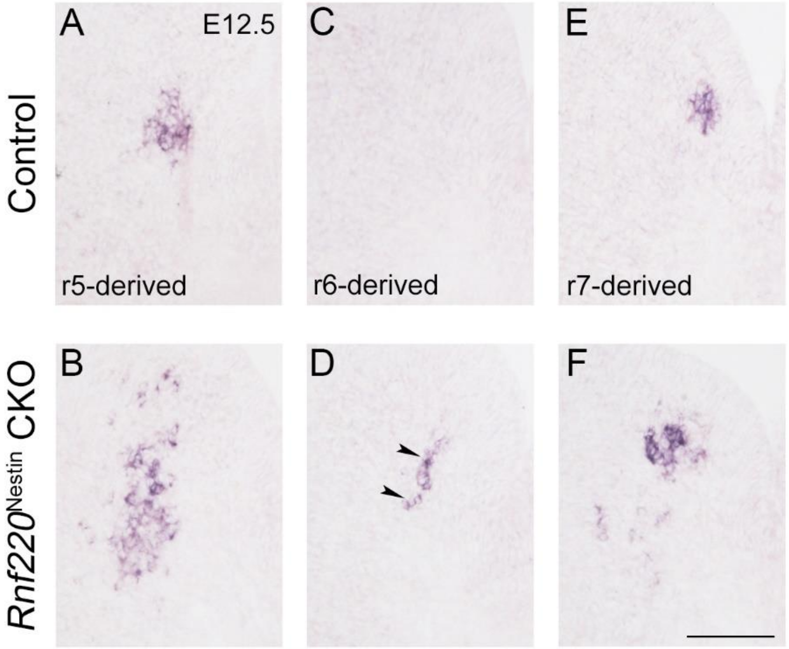

Figure S3. Ectopic expression of *Hb9* in the Hindbrain of *Rnf220*^{Nestin} CKO embryos at E12.5.

(A-F) *In situ* hybridization for *Hb9* on the sections of hindbrain of *Rnf220*^{Nestin} CKO (A, C and E) and control (B, D and F) embryos.

(A, B) The expression of *Hb9* is increased in *Rnf220*^{Nestin} CKO embryos (B) at r5-derived level compared with controls (A).

(C, D) Ectopic expression of *Hb9* occurred in *Rnf220*^{Nestin} CKO embryos (D, arrowheads) at r6-derived level whereas no somatic motor neuron (sMN) exists at this level in controls (C).

(E, F) More *Hb9*$^{+}$ sMNs are observed in *Rnf220*^{Nestin} CKO embryos (F) at r7-derived level than in controls (E). Scale bar, 100μm (F; also applies to A-E).

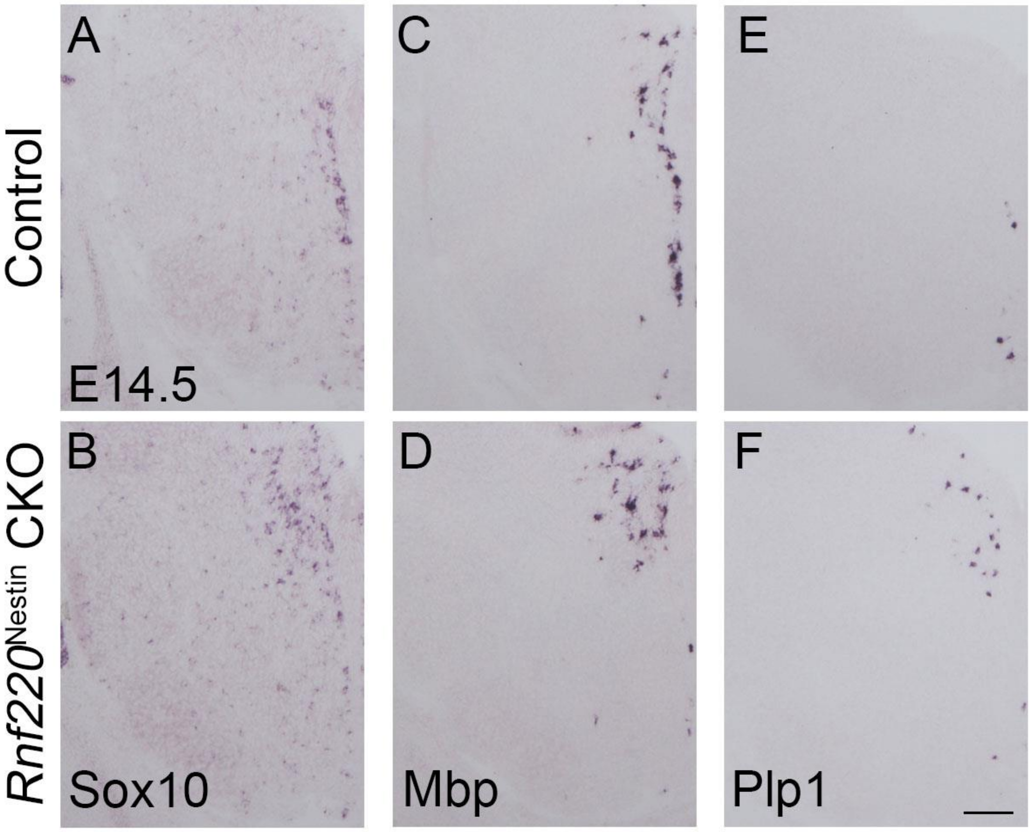

Figure S4. Abnormal distribution of mature oligodendrocytes in the *Rnf220*^{Nestin} CKO hindbrain at E14.5.

(A-F) *In situ* hybridization for *Sox10* (A and B), *Mbp* (C and D) and *Plp1* (E and F) shows abnormal distribution of mature oligodendrocytes in *Rnf220*^{Nestin} CKO (B, D and F) than controls (A, C and E) at E14.5. Scale bar, 100μm (F; also applies to A-E).
